# Heartburn Center Set-Up in a Community Setting: Engineering and Execution

**DOI:** 10.3389/fmed.2021.662007

**Published:** 2021-11-10

**Authors:** Atul Maini, John Sun, Borys Buniak, Stacey Jantsch, Rachel Czajak, Tara Frey, B. Siva Kumar, Amarpreet Chawla

**Affiliations:** ^1^The Heartburn Center at St. Joseph's Health, Liverpool, NY, United States; ^2^Johns Hopkins University, Baltimore, MD, United States

**Keywords:** gastroesophageal reflux disease (GERD), center of excellence (CoE), heartburn center (HBC), quality of life (HRQL), length of stay (LOS)

## Abstract

**Background:** Optimal management of gastroesophageal reflux disease (GERD) requires a concerted team of physicians rather than an individual approach. While an integrated approach to GERD has previously been proposed, the practical execution of such a “center of excellence” (COE) has not been described, particularly in a community setting. Ranging from initial consultation and diagnosis to surgical intervention for complex disease, such an approach is likely to provide optimal care and provide surveillance for patients with a complex disease process of GERD.

**Methods:** We report our approach to implement an integrated heartburn center (HBC) and our experience with the first cohort of patients. Patients treated in the HBC were followed for 2 years from initial consultation to completion of their appropriate treatment plan, including anti-reflux surgery. The performance prior to the HBC set-up was compared to that post-HBC. Performance was measured in terms of volume of patients referred, referral patterns, length of stay (LOS), and patient health-related quality of life (HRQL) pre- and post-surgery.

**Results:** Setting up the HBC resulted in referrals from multiple avenues, including primary care physicians (PCPs), emergency departments (EDs), and gastroenterologists (GIs). There was a 75% increase in referrals compared to pre-center patient volumes. Among the initial cohort of 832 patients presenting to the HBC, <10% had GERD for <1 year, ~60% had GERD for 1–11 years, and ~30% had GERD for ≥12 years. More than one-quarter had atypical GERD symptoms (27.6%). Only 6.4% had been on PPIs for <1 year and >20% had been on PPIs for ≥12 years. Thirty-eight patients were found to have Barrett's esophagus (4.6%) (up to 10 times the general population prevalence). Two patients had dysplasia. Seven patients (0.8%) received radiofrequency ablation (RFA) for Barrett's esophagus and two patients received endoscopic mucosal resection (EMR) for Barrett's esophagus-related dysplasia. The most common comorbidities were chronic pulmonary disease (16.8%) and diabetes without complications (10.6%). Patients received treatment for newly identified comorbid conditions, including early maladaptive schemas (EMS) and generalized anxiety disorder (GAD) (*n* = 7; 0.8%). Fifty cases required consultation with various specialists (6.0%) and 34 of those (4.1%) resulted in changes in care. Despite the significant increase in patient referrals, conversion rates from diagnosis to anti-reflux surgery remained consistent at ~25%. Overall HRQL improved year-over-year, and LOS was significantly reduced with potential cost savings for the larger institution.

**Conclusions:** While centralization of GERD care is known to improve outcomes, in this case study we demonstrated the clinical success and commercial viability of centralizing GERD care in a community setting. The integrated GERD service line center offered a comprehensive, multi-specialty, and coordinated patient-centered approach. The approach is reproducible and may allow hospitals to set up their own heartburn COEs, strengthening patient-community relationships and establishing scientific and clinical GERD leadership.

## Introduction

Gastroesophageal reflux disease (GERD) is a complex disease process that affects ~20% of adults in the US (1, 2). Many patients with GERD self-medicate or are treated with medication for years without proper work-up or evaluation by a physician. Patients with persistent and intense symptoms of GERD (i.e., refractory GERD) experience decreased health-related quality of life (HRQL) and lower productivity at work and in daily activities, potentially resulting in higher healthcare and employer costs (3). Persistent reflux symptoms, despite medical therapy, have been associated with debilitating psychological comorbidities including mental health disorders, sleep disorders, and psychological distress to patients (4–6). GERD has also been shown to be associated with medical comorbidities including hypertension, diabetes, hyperlipidemia, coronary heart disease, alcohol-related illness, stroke, obesity, chronic obstructive pulmonary disease, asthma, biliary stone, anxiety, depression, chronic kidney disease, and cirrhosis (7, 8). GERD can also have significant potential complications such as dysphagia, erosive esophagitis, Barrett's esophagus, and esophageal adenocarcinoma (9).

The effective treatment of GERD patients requires an awareness of the clinical spectrum of GERD and its varied symptomatology, associated comorbidities, and potential complications. Despite the prevalence and impact of GERD, the patient and treatment pathways for a considerable number of patients with GERD are suboptimal (10, 11). Patients with GERD may present with typical and/or atypical symptoms that may be missed if not appropriately recognized, diagnosed, and treated. Due to the association of GERD with many psychological and medical comorbid conditions, screening and treatment of GERD may aid as a predictor and allow for earlier detection and treatment of undiagnosed comorbid diseases. However, referral pathways for patients with GERD from primary care physicians (PCPs) to specialists are fragmented and not adequately organized, thus affecting the process of patient evaluation, treatment, continuity of care, and ultimately clinical outcomes and costs (12).

Centers of excellence (CoEs) are specialized programs that supply high concentrations of expertise and related resources centered on particular medical areas and delivered in a comprehensive, interdisciplinary fashion (13). CoEs provide an opportunity to align physicians in quality improvement, reduce costs through greater efficiencies, and create market differentiation through clinical excellence and high patient satisfaction (14–18). CoEs also have the ability to dramatically enhance the depth and breadth of healthcare services available in communities that otherwise would not have had access to such exceptionally high levels of quality care (13).

In order to optimize treatment options for GERD, the concept of a “Heartburn Center” as a CoE in foregut disease is emerging as our understanding of the management of the array of esophageal diseases continues to grow (19). An integrated and collaborative multi-specialty approach to the management of referral, patient, and treatment pathways is designed to provide a comprehensive pathway of care for GERD by improving communication, education, and compliance with testing and treatment. A CoE approach has been associated with improved healthcare economics and patient outcomes (1, 20).

While significant strides have been made in defining the quality of GERD care in an integrated CoE (19), the question remains as to what the underlying components in successfully executing those best practices in such a center would be. As such, the purpose of this study was to describe our approach to implement a heartburn center (HBC) at our community-based facility, report potential challenges and barriers in setting up and running these centers and steps to mitigate them, and most importantly, provide a reproducible methodology for the design and development of a functional HBC with the aim to have hospital administrators and healthcare professionals identify practices, tools, and resources needed to set up their comprehensive heartburn CoE.

## Materials and Methods

**Figure 1:**
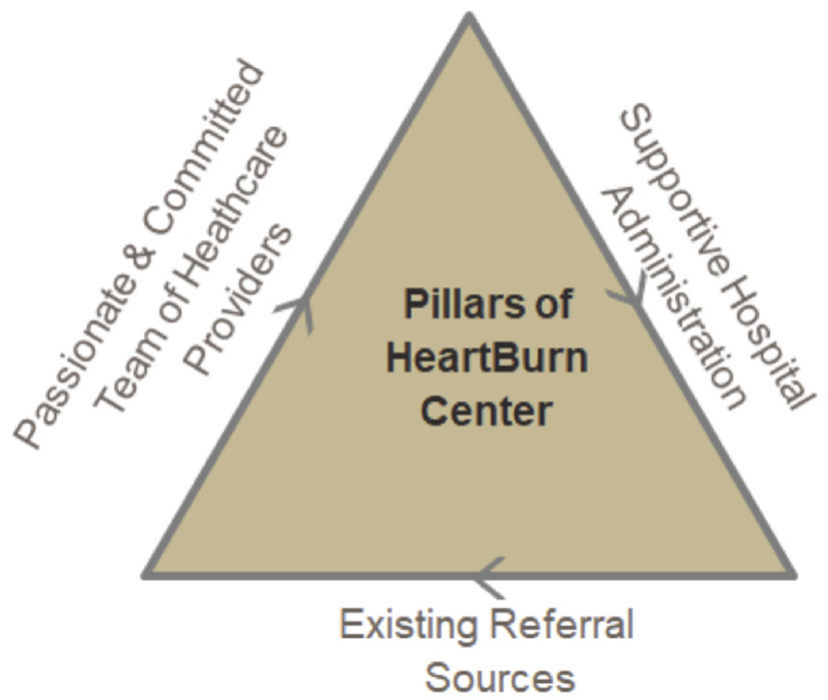
Development of a new practice model for heartburn center of excellence.

The concept of the interdisciplinary practice model was first introduced to our hospital leadership team in early 2016 by a surgeon and GI. St. Joseph's Physicians Surgical Services, part of St. Joseph's Hospital Health Center, offers comprehensive surgical care for ambulatory, elective and critically ill patients. Surgical services range from oncology, bariatric, including both laparoscopic and robotic surgeries. As illustrated in Figure 1, a successful heartburn CoE is supported by three key elements: a team of healthcare providers, hospital administration, and referral sources.

### Referral Sources

**Figure 2:**
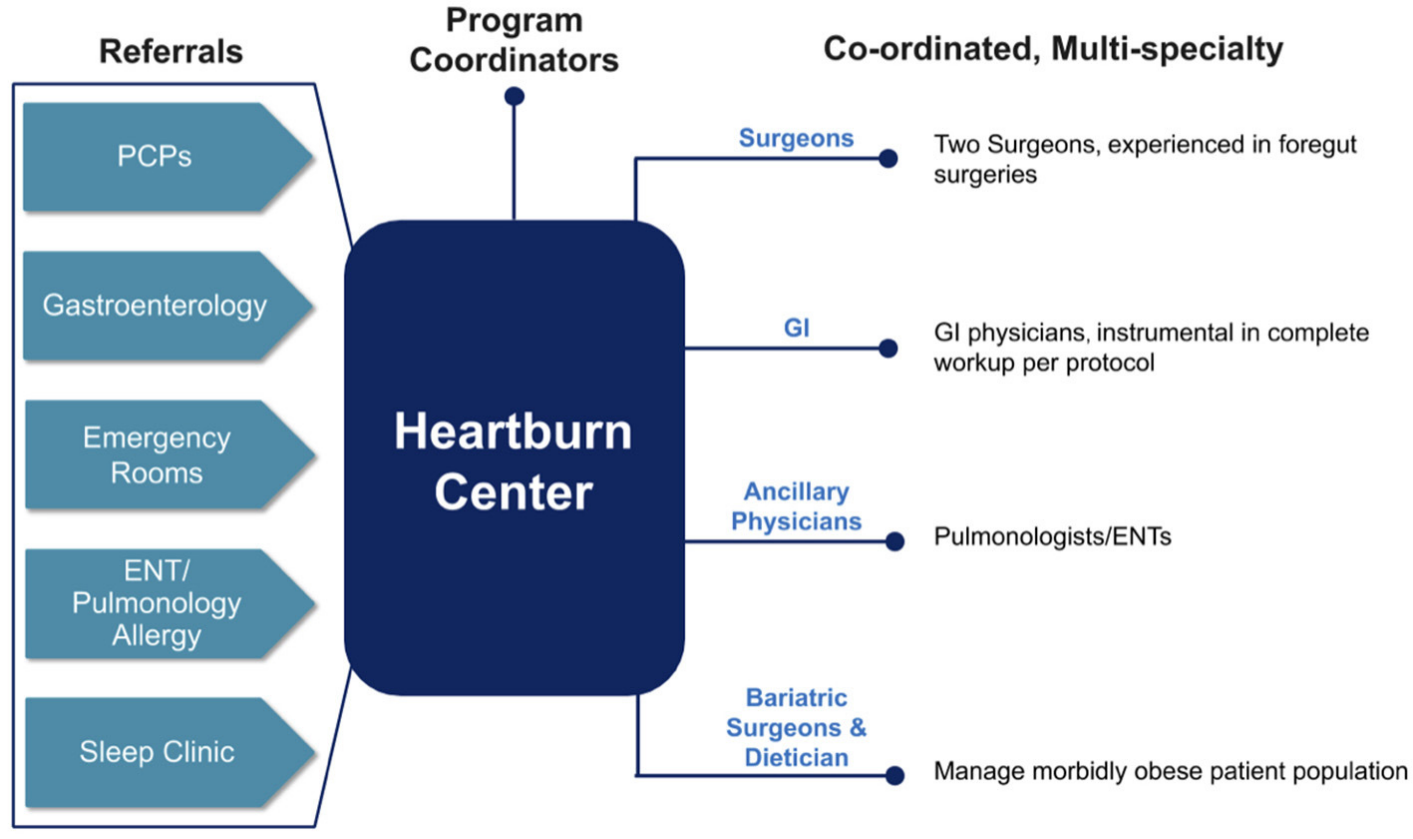
Organizational structure of the practice model for our heartburn center of excellence.

To provide optimal patient care, it is critical that evidence-based multi-disciplinary referral pathways are set up that include but are not limited to patients, PCPs, GIs, ear, nose, and throat physicians (ENTs), pulmonary physicians, emergency physicians, and surgeons. Figure 2 demonstrates the integrated organization structure in place at our HBC.

GIs should be a part of the core group and should be involved early in the patient's treatment decision. A previous administrative claims database analysis that examined healthcare resource utilization of patients with a diagnosis of GERD found that esophageal objective testing was inadequate when patients were not referred to GIs, and furthermore, timely transfer of GERD patients to GIs when empiric treatment is insufficient may lead to improved clinical management (21). It is our estimate that in our HBC, as many as 20% of patient referrals may have been lost if GIs were not integrated into the HBC. However, to ensure timely referrals and to avoid referrals from GIs that are only limited to those patients with long-term, severe symptoms and large hiatal hernias, improved education for recognizing symptoms suggestive of GERD, and consequently to appropriately channel patients through diagnostic and treatment pathways (22).

### Hospital Administration

An optimized program has well-coordinated milestones of timely consultation, diagnostic workup, a documented treatment plan, and regular follow-up.

#### Consultation

For patients to be funneled into the center, we developed a patient outreach program that facilitated patient flow, importantly with dedicated program coordinator and director. Such a program ensured patient education about the center's value proposition and differentiation from other similar centers in the region. Centers may consider developing a hub to receive dedicated calls from potential patients and appropriate referrals. Following referral, it is critical to set outcome expectations with the patient and expectations of downstream effect.

#### Patient Navigation Platform

A digital platform that facilitates patient progress tracking throughout their continuum of care and also facilitates comprehensive data collection regarding the effectiveness of such a center is vital for a service line center. An integrated implementation of such a platform with the Epic electronic health record (Epic Corporation, Madison, WI) system at our center facilitated referrals from primary care and ED. Especially patients who came to the ED with chest pain and who were typically sent back if no cardiac involvement was determined, are now being regularly referred to the HBC with improved disease awareness of atypical symptoms of GERD as well as the facility to seamlessly refer those patients to the HBC.

#### Diagnostic Work-Up

Having a protocol for appropriate identification of patients is important. Esophageal objective testing is required to anatomically and physiologically evaluate the presence and progression of GERD in patients being considered for surgery. Founded on evidence- and experienced-based consensus (23, 24), we established a robust diagnostic protocol that included endoscopy, ambulatory pH monitoring, manometry, and evaluation of LES length and degree of hiatal herniation by EGD for evaluating the patient's condition and to rule out other diagnoses such as malignancies, achalasia, and scleroderma (25). At our center, we combined the results of all the diagnostics to document the presence and extent of the disease and assist in planning the surgical approach.

#### Treatment Plan

We set up a treatment protocol for every patient prior to the start of disease management. We have described the selection criteria in our manuscript which is based on patients Symptoms, EMS study, pH study, Endoscopy, Barium swallow study and GAD-Generalized Anxiety score and patient preference. Our protocol dictates that post-diagnostic workup, the patient be offered treatments based on the pathology and to rule out other intra-abdominal pathologies such as gallbladder or peptic ulcer disease that may be potentially misdiagnosed as reflux disease. Patients who are candidates for anti-reflux surgery are given options to undergo either a hiatal hernia repair with magnetic sphincter augmentation device implant/LINX® procedure or a robotic hiatal hernia procedure with a posterior robotic Toupet fundoplication (RTF). Patients with a BMI >35 are first educated about lifestyle modification including weight loss or referred to a bariatric surgeon if surgery is indicated. Patients with any anxiety causing esophageal symptoms are referred to a gut psychologist. Importantly, recurring clinical meetings and grand rounds are setup with core physicians to discuss an optimal treatment plan for each patient.

#### Follow-Up

The patients in the center are typically followed at 2, 8 weeks, 6 months and 1 year in the postoperative period in patients with LINX® procedure and fundoplications. The patients are surveyed and their HRQL scores are calculated at each visit.

### Team of Healthcare Providers

Central to the HBC is the surgeon and GI partners. It is critical that the surgeons have foregut surgery experience and that the GIs are experienced in foregut physiology and pathology and are willing to follow established pre-surgery diagnostic pathways including manometry and pH testing, understand the US payer environment, and importantly have demonstrated history of collaborating with administration and colleagues.

Ancillary physicians may include a gut psychologist, pulmonologist, ENT specialists, bariatric surgeon, and nutritionist. A supportive staff of healthcare providers should include a program coordinator, nurse practitioners, physician assistants, and a nutritionist. Finally, it is important to partner with the hospital administration such as a program director who has the authority to approve new programs or looking to establish leadership in a particular disease area.

### HBC Performance Measurement: Methodology

**Figure 3:**
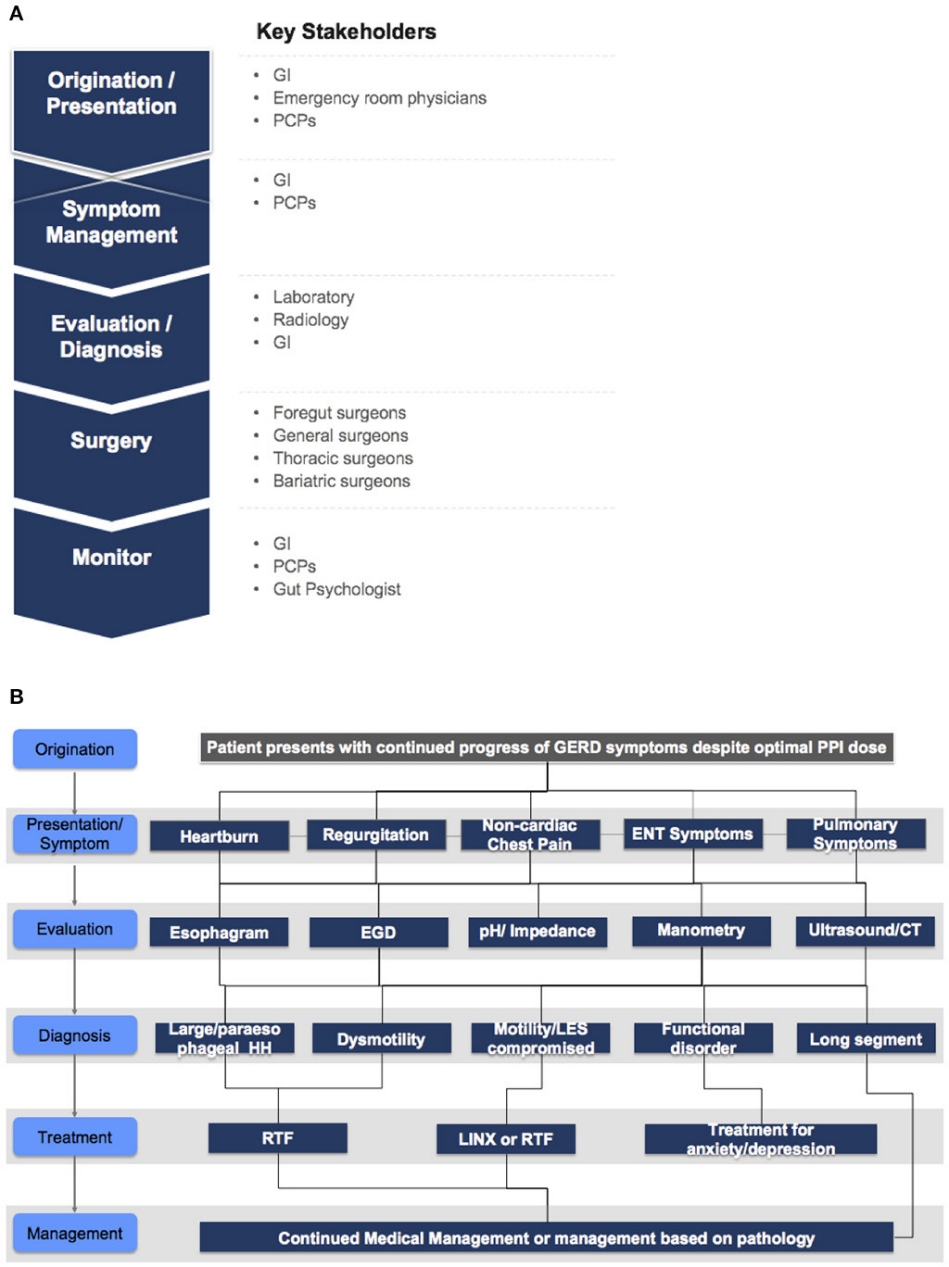
**(A)** Patient flow and associated providers. **(B)** Treatment decision tree implemented at our heartburn center.

We followed patients over 2 years from initial consultation to completion of an appropriate treatment plan. Figures 3A,B illustrates the patient flow protocol and treatment decision tree that were implemented at our HBC, respectively. Retrospective data at each node of patients' treatment pathway were collected and analyzed. Specifically, four endpoints were assessed and quantified: (1) Patient volumes and corresponding conversion rates through consult-diagnostics-treatment; (2) Referral patterns and sources of referral; (3) Trends on length of stay, and finally; (4) Patient outcomes in terms of HRQL impact.

#### Inclusion Criteria

The study included patients who were 21 years of age and older, had a history of typical or atypical GERD symptoms, at least 8 weeks of once or twice daily PPI therapy with or without breakthrough symptoms, actively seeking alternative surgical treatment options for bothersome GERD symptoms including, but not limited to heartburn, regurgitation, and dysphagia. Subjects were willing and able to cooperate with follow-up examinations. For those having LINX® implanted, the LINX® Instruction for Use (IFU) (26) was additionally followed as the inclusion criteria.

#### Exclusion Criteria

Subjects with suspected or known allergies to titanium, stainless steel, nickel, or ferrous materials, esophageal dysmotility, connective tissue disorders, or severe anxiety symptoms.

## Results

### HBC Assessment and Metrics

Tables 1, 2 present the baseline demographic and clinical characteristics of the first 832 patients who presented to the HBC. Approximately one-third of patients were younger than 45, one-fifth were older than 65, and 45.2% were between 46 and 65 years of age. Six out of 10 patients were female and most (83.1%) were Caucasian (7.1% Black or African American, 5.0% other, 3.1% Hispanic, and 1.7% Asian). Half of the patients had commercial insurance (49.4%) and the remainder had Medicaid (28.2%) or Medicare (22.4%).

**Table 1:** Baseline demographic characteristics of patients presenting to the HBC.

**Patient demographic characteristic/comorbidity**	***N***	**%/mean (SD)**
All	832	100%
Age		
<19	2	0.2%
19 to 25	39	4.7%
26 to 45	247	29.7%
46 to 65	378	45.4%
> 65	166	20.0%
Gender		
Female	500	60.1%
Male	332	39.9%
Race		
Caucasian	691	83.1%
Black or African American	59	7.1%
Asian	14	1.7%
Hispanic	26	3.1%
American Indian/Alaska Native	0	0.0%
Other	42	5.0%
Unknown/Refused	0	0.0%
Health insurance payer		
Medicaid/Molina/Fidelis/Government plans	235	28.2%
Commercial	411	49.4%
Medicare	186	22.4%

**Table 2:** Baseline clinical characteristics of patients presenting to the HBC.

**Patient demographic Characteristic/Comorbidity**	***N***	**%/mean (SD)**
All	832	100%
Body mass index (BMI)		
<18.5 (Underweight)	6	0.7%
18.5–24.9 (Normal)	165	20.2%
25.0–29.9 (Overweight)	266	32.6%
30.0–<35.0 (Obesity Class I)	219	26.8%
35.0–<40.0 (Obesity Class II)	99	12.1%
40.0+ (Obesity Class III)	62	7.6%
Charlson comorbidity score		
0	469	56.4%
1–2	265	31.9%
3–4	80	9.6%
≥5	18	2.2%
Comorbidities		
Myocardial infarction	40	4.8%
Congestive heart failure	12	1.4%
Peripheral vascular disease	35	4.2%
Cerebrovascular disease	32	3.8%
Chronic pulmonary disease	140	16.8%
Connective tissue disease-rheumatic disease	38	4.6%
Peptic ulcer disease	15	1.8%
Mild liver disease	17	2.0%
Diabetes without complications	22	2.6%
Diabetes with complications	88	10.6%
Paraplegia and hemiplegia	12	1.4%
Renal Disease	52	6.3%
Moderate or severe liver disease	51	6.1%
Type of symptoms		
Typical	602	72.4%
Atypical	230	27.6%
Duration of GERD (years)		
<1	71	8.5%
1–3	153	18.4%
4–7	187	22.5%
8–11	160	19.2%
12–15	80	9.6%
15+	181	21.8%
Duration Medication Use (PPI/H2/Other/Combo) (years)		
<1	53/28/2/7	6.4/3.4/0.2/0.8%
1–3	91/21/2/20	10.9/2.5/0.2/2.4%
4–7	100/19/4/22	12.0/2.3/0.5/2.6%
8–11	96/10/2/19	11.5/1.2/0.2/2.3%
12–15	50/5/3/15	6.0/0.6/0.4/1.8%
15+	124/11/9/30	14.9/1.3/1.1/3.6%
Barrett's Esophagus		
Short segment	33	4.0%
Long segment	5	0.6%
Dysplasia	2	0.2%

Approximately one in five patients had normal weight, one-third were overweight, and the remainder (46.5%) were obese (Table 2). More than half of patients had a Charlson Comorbidity Index (CCI) score of 0, nearly one-third had a CCI of 1–2, nearly 10% had a score of 3–4, and only 2.2% had a score of 5 or greater. The most common comorbidities were chronic pulmonary disease (16.8%) and diabetes without complications (10.6%) (Table 2). Patients received treatment for any newly identified comorbid conditions, including early maladaptive schemas (EMS) and generalized anxiety disorder (GAD) (*n* = 7; 0.8%) (data not shown). In total, 50 of the 832 cases required conference/consultation with various specialists (6.0%) and 34 of those (4.1%) resulted in changes in care. Seventeen of the cases (3.0%) resulted in changes in medical management and 17 (3.0%) resulted in a change in the type of recommended GERD surgery (data not shown).

More than one-quarter of patients with GERD had atypical symptoms (27.6%). <10% of patients had GERD for <1 year, ~60% had GERD for 1–11 years, and 3 in 10 patients had GERD for 12 or more years. Only 6.4% of patients had been on PPIs for less than a year and more than 20% of patients had been on PPIs for 12 or more years. Thirty-eight patients had Barrett's esophagus (4.6%) (Table 2), which is up to 10 times the prevalence rate found in the general population (27). Two patients were found to have dysplasia. Seven patients (0.8%) received radiofrequency ablation (RFA) for Barrett's esophagus and two patients received endoscopic mucosal resection (EMR) for Barrett's esophagus-related dysplasia (data not shown).

Trends in the performance of the HBC were assessed over 2 years, specifically 2017–2019, and benchmarked across four different metrics: referrals, patient volumes, length of stay, and HRQL.

### Referrals Patterns and Sources

The primary source of referrals were self-referrals (patients from advertisements, television events, print, public patient seminars (24%), GI (20%), primary care centers (54%), with others from emergency department (ED) and specialty services. Most notably, GIs were a *de novo* referral source that were unavailable to our institution before the setup of the HBC.

These patients were distributed across 79 zip codes around the HBC. Although there are hospitals in this catchment area that are larger than ours in terms of patient volumes, our HBC is capturing a larger share of the GERD patients due to our integrated care approach for these patients.

### Patient Volumes

**Figure 4:**
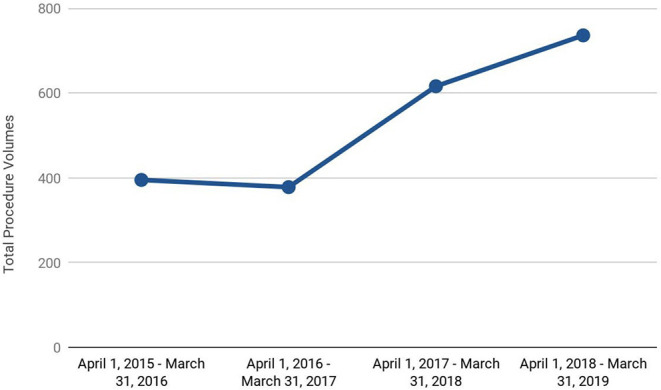
Upward trend in total anti-reflux procedure (pre-surgery diagnostics and surgical) volumes in our heartburn center.

As illustrated in Figure 4, there was an upward trend in patient volumes to the HBC year-over-year. Specifically, compared to pre- HBC, patient volumes increased by 75% post-HBC.

The uptick in volumes was across the board including pre-surgery diagnostics as well as anti-reflux surgical procedures. Importantly, an integrated approach provided the larger institution an opportunity to adopt novel surgical procedures such as LINX®, as well as non-surgical endoscopic procedures such as Radiofrequency Ablation, Endoscopic Mucosal Resections that were not performed prior to the start of the HBC.

**Figure 5:**
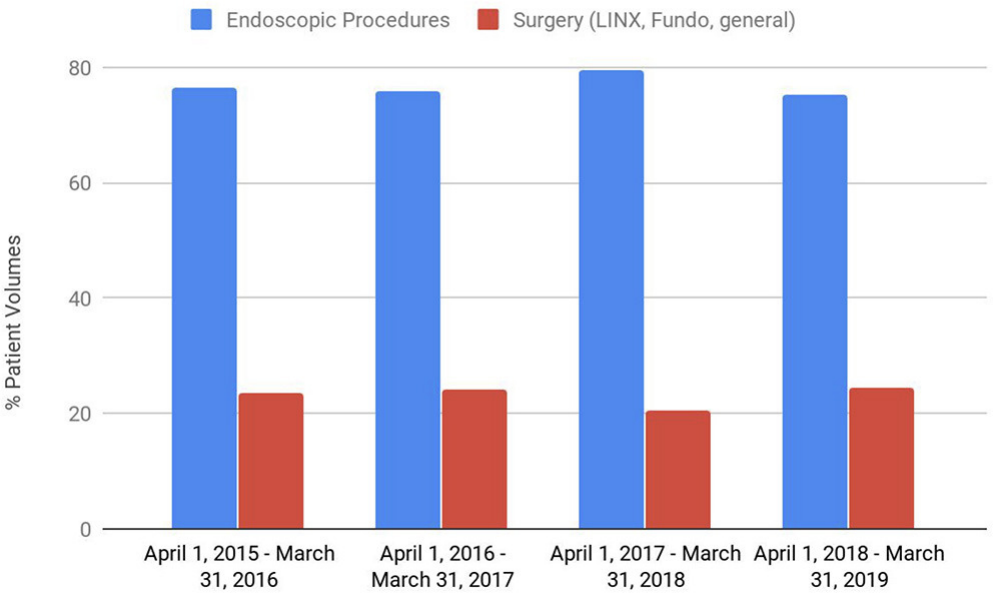
Patient volumes as a percentage of the total pre-surgery diagnostics and surgical volumes.

As depicted in Figure 5, even as the patient volumes have increased, the conversion rates from diagnostics to surgery have remained consistent in part due to our protocol-driven approach and management of the disease.

### Length of Stay

**Figure 6:**
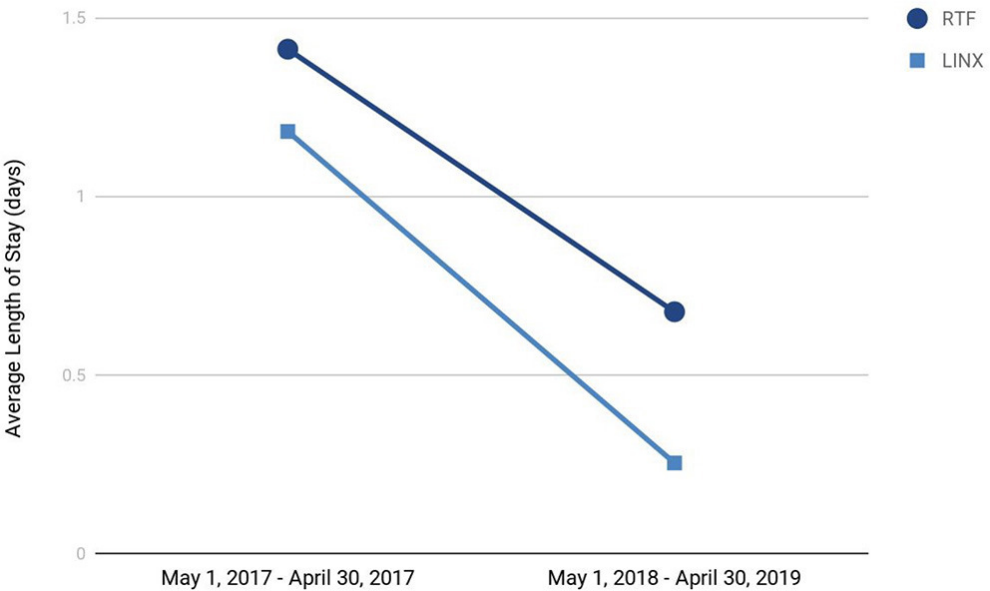
Trends in average length of stay for the two primary anti-reflux surgeries conducted at our heartburn center.

The length of stays (LOS) for the two important anti-reflux procedures, LINX® and RTF, were recorded. As illustrated in Figure 6, as we gained more experience with these patients, the LOS reduced dramatically by over 50% over 2 years. The reduction in LOS resulted in significant economic benefit to the hospital.

### Health-Related Quality of Life (HRQL)

**Figure 7:**
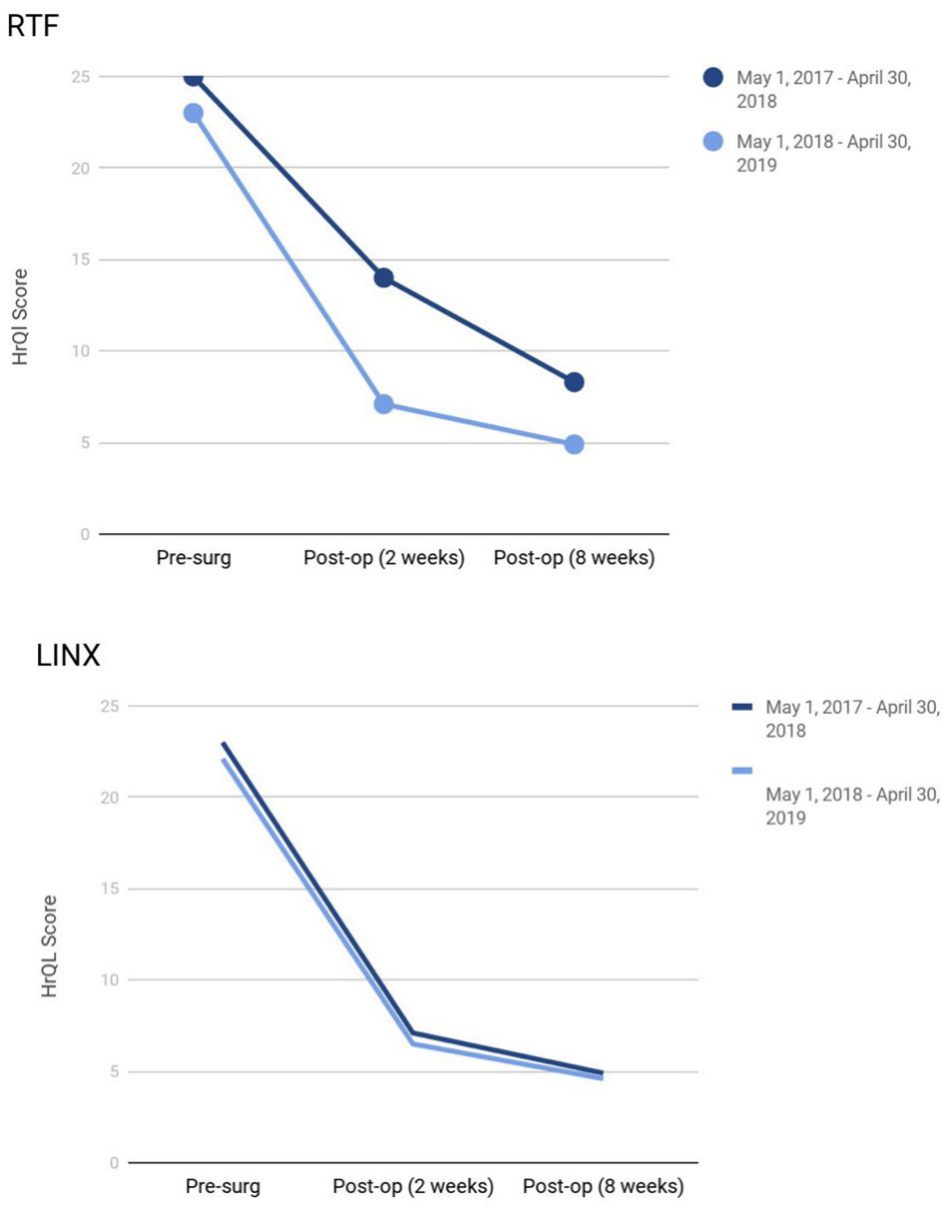
Two-year trends in HRQL scores for patients undergoing LINX® and RTF.

Patients implanted with LINX® as well as those who underwent RTF demonstrated a statistically significant improvement in GERD-HRQL score at 8 weeks compared to baseline: An average score of 24 (range 36–0) vs. 6 (range 28–0) in LINX® patients and 23 (range 41–0) vs. 5 (range 23–0) in the RTF population. As illustrated in Figure 7, while HRQL consistently improved post-surgery in both the populations, there was no significant difference in the HRQL improvement year-over-year.

## Discussion

An integrated GERD service line center offers a comprehensive, multi-specialty, and coordinated patient-centered approach. Ranging from initial consultation and diagnosis to surgical intervention for complex disease, such an approach is likely to provide optimal care and provide surveillance for patients for a complex disease process of GERD.

In this article, we reported our approach to a practice model of an integrated HBC based on our experience at our facility. The integrated approach drove quality and efficiency in terms of coordinated care that enabled care cross-over and efficient resource utilization. Furthermore, setting up treatment protocols and defining patient pathways standardized the workflow and provided a definition of the center. Importantly, local outreach and marketing helped develop interpersonal relationships with referral physicians resulting in recognition of the center as a “Real Antireflux Heartburn Center.” That in turn promoted specialization and differentiation from competition, leading to superior results for our HBC both in terms of patient volumes and patient care. Following an audit of the retrospective data presented here, the regional Excellus BlueCros BlueShield now considers the LINX® device implant a medically necessary procedure and provides insurance coverage to its 1.5 million members (26). Challenges remain to manage obese patients with GERD, the hospitals controlling their patients to be referred across the hospital systems, acceptance by the primary care and even some GI physicians to consider GERD to be a surgically manageable disease. The limitations of our study is that it is a single center effort and since we were not able to find other centers of similar integrity in a community set up, we were unable to compare our results with other centers. The community hospitals will need to establish similar set up to address disease of such complex nature to provide best care to the patients. Further discussions and dialogues are needed. We believe that the integrated approach not only provides optimal patient care and satisfaction, but also a unique opportunity for the institution to grow and adapt novel technologies.

## Data Availability Statement

The raw data supporting the conclusions of this article will be made available by the authors, without undue reservation.

## Author Contributions

All authors listed have made a substantial, direct and intellectual contribution to the work, and approved it for publication.

## Conflict of Interest

The authors declare that the research was conducted in the absence of any commercial or financial relationships that could be construed as a potential conflict of interest.

## Publisher's Note

All claims expressed in this article are solely those of the authors and do not necessarily represent those of their affiliated organizations, or those of the publisher, the editors and the reviewers. Any product that may be evaluated in this article, or claim that may be made by its manufacturer, is not guaranteed or endorsed by the publisher.
